# Lateralized occipito-temporal N1 responses to images of salient distorted finger postures

**DOI:** 10.1038/s41598-017-14474-x

**Published:** 2017-10-26

**Authors:** Miguel G. Espírito Santo, Hsin-Yuan Chen, Martin Schürmann

**Affiliations:** School of Psychology, University of Nottingham, NG7 2RD Nottingham, UK

## Abstract

For humans as social beings, other people’s hands are highly visually conspicuous. Exceptionally striking are hands in other than natural configuration which have been found to elicit distinct brain activation. Here we studied response strength and lateralization of this activation using event-related potentials (ERPs), in particular, occipito-temporal N1 responses as correlates of activation in extrastriate body area. Participants viewed computer-generated images of hands, half of them showing distorted fingers, the other half showing natural fingers. As control stimuli of similar geometric complexity, images of chairs were shown, half of them with distorted legs, half with standard legs. The contrast of interest was between distorted and natural/standard stimuli. For hands, stronger N1 responses were observed for distorted (vs natural) stimuli from 170 ms post stimulus. Such stronger N1 responses were found for distorted hands and absent for distorted chairs, therefore likely unrelated to visuospatial processing of the unusual distorted shapes. Rather, N1 modulation over both hemispheres – but robustly right-lateralized – could reflect distorted hands as emotionally laden stimuli. The results are in line with privileged visual processing of hands as highly salient body parts, with distortions engaging neural resources that are especially activated for biological stimuli in social perception.

## Introduction

Distorted postures of body parts that, for example, might occur following an accident are highly salient stimuli in social perception and can make an observer feel uneasy. Does this exceptional salience reflect particular patterns of distortion-related processing in the observer’s brain? In the first study to address this question, Avikainen *et al*.^1^ chose computer-generated images of hands with distorted finger postures as stimuli. Hands are particularly conspicuous body parts because they reveal an observed person’s actions and intentions. Avikainen *et al*.^1^ identified distortion-related lateral-occipital activation, starting from 260 ms after stimulus onset, using magnetoencephalography (MEG) as a method with high temporo-spatial resolution^1,2^. The latency suggests amygdalar feedback to the occipital cortex, in line with participants’ reports that they felt uneasy watching the distorted hand stimuli. Beyond lateral-occipital cortex, further areas were found activated during processing of distortions in a functional magnetic resonance imaging (fMRI) study^3^. Due to the pattern of activation – including sensorimotor cortex, postcentral somatosensory areas, amygdala, and insula – a mechanism of embodied perception was suggested as underlying the exceptional salience of distorted hand and finger postures^3^.

The current study aims to assess the role of lateral-occipital brain areas as nodes in the network of distortion-sensitive brain areas^1,3^, using an electrophysiological method to determine timing of activation. Lateral occipital and fusiform areas are sensitive to images of body parts, according to fMRI studies (extrastriate body area, EBA^4^; fusiform body area, FBA^5^; for review see^6^). Partly overlapping with EBA, areas of lateral occipital cortex show activation that is sensitive to images of hands (vs other body parts^7,8^), or to hand movement^9^. Complementary with these fMRI results on occipito-temporal areas supporting social perception, electrophysiological methods such as MEG or EEG have provided information about response timing. In event-related EEG potentials (ERPs) from occipito-temporal electrodes, face-sensitive responses have been identified at N1 latency. Images of faces elicit strong N1 responses peaking at 170 ms (N170^10^) although the selectivity of this response is under debate^11–15^. Separable from face-sensitive responses, N1 modulations have been identified as a response to whole bodies (vs faces^16^; vs faces, scenes, objects and peaking at 190 ms^17^). Body-sensitive responses from lateral occipital cortex have been replicated in numerous EEG studies, although some of these report earlier N1 latencies than 190 ms (for example^18^ 176 ms, collapsed across whole bodies and body parts such as hands, and^19^ 164 ms in healthy control group).

**Figure 1:**
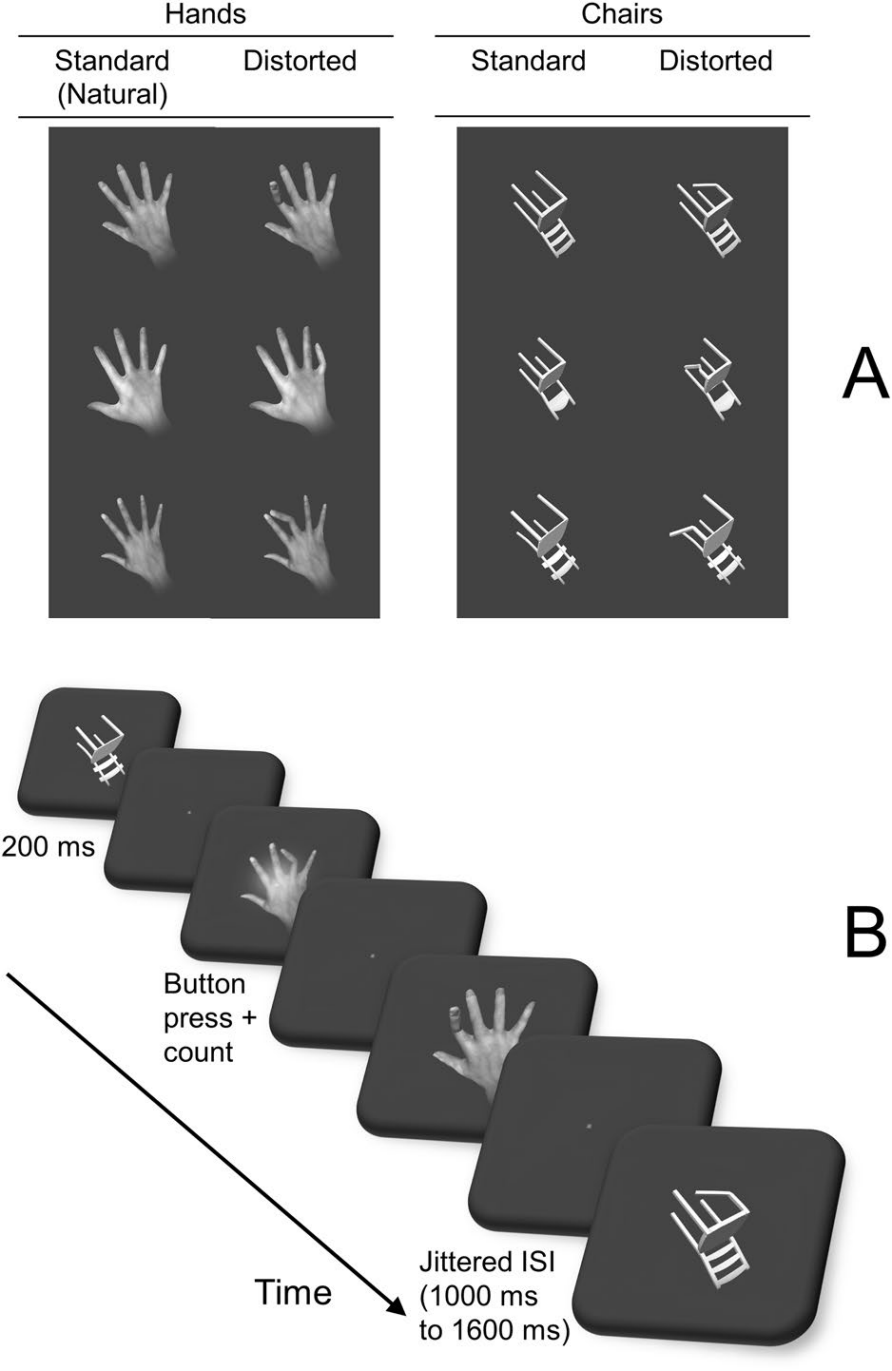
Stimulus setup. (**A**) Stimulus categories, 3 (out of 6) exemplars per category, each in natural and distorted configurations. Stimuli computer-generated using an in-house protocol (developed by M.G.E.S, see Methods and Supplementary Information). (**B**) Stimulus timeline. Stimuli not shown to size (actual size 4° of visual field).

N1 responses to faces (and corresponding waveforms in MEG) vary with emotional expression of faces (for review, see^20^). Likewise, fearful whole-body postures modulate body-sensitive ERPs, although it is controversial whether the modulation occurs at N1 latency^21^ or at other ERP peaks, leaving N1 unaffected^22^. Although of a different quality than fearful body postures, images of distorted hands and fingers are emotionally laden, in that they can make an observer feel uneasy. This emotional quality could lead to stronger N1 responses as could be predicted from MEG^1^ and fMRI results^3^ with similar stimuli. However, stronger N1 responses for distorted (relative to natural) hands could also be due to the novelty of the distorted hands which require increased visuospatial processing of the 2D-presented 3D models. To separate between emotional contents vs novelty and visuospatial processing as underlying stronger N1 responses, it is necessary to introduce additional control stimuli. This extended approach goes beyond earlier studies wherein all analyzed responses were either to distorted or to natural hands and fingers because these studies aimed to facilitate detection of distortion-related activation^1,3^. Suitable control stimuli need to be matched in terms of novelty and visuospatial processing while lacking the emotional contents of distortion applied to hands as biological stimuli. Therefore, non-biological control stimuli of similar geometric complexity as hands are needed. In fMRI studies in search of activation sensitive to images of hands, chairs served as non-biological controls^7,8,23^. In certain orientations, back of the hand and four fingers (except thumb) are compellingly similar to the backrest and four legs of a chair. Here we introduce a novel category of control stimuli, closely matched to distorted hands, namely chairs with distorted legs (“distorted chairs”). Figure 1 gives examples of all stimulus categories.

Building on earlier research reviewed above^1,3^, the first research question of our current study is whether distortion-sensitive responses can also be found in EEG and/or ERPs, a method which is more widely available than MEG as used by Avikainen *et al*.^1^. To increase the sensitivity of the EEG analysis, the search can be limited in terms of electrode locations and response latency windows. The waveform of interest is the occipito-temporal N1 response with sensitivity for whole bodies (at 190 ms^17^) and/or body parts (at 170 ms^18^), linked to EBA activation. One further way of increasing the sensitivity of EEG analysis is to keep the number of conditions minimal, thereby ensuring a sufficiently large number of trials per condition. Therefore, unlike the earlier fMRI study with hand stimuli in first-person and third-person perspective, the current EEG study shows all hand stimuli in first-person perspective which elicited largest responses in the earlier fMRI study^3^. Our second research question is whether distortion-related N1 responses (if any) are lateralized. Due to bilateral but strongly right-lateralized sensorimotor activation to distorted hands in fMRI^3^, it is of interest whether asymmetry (right stronger than left) occurs at the early latency of N1 responses. Comparison of ERPs across conditions requires subjects’ attention to visual stimuli throughout the experiment. Therefore, subjects were instructed to keep mental counts of a stimulus feature (shadow superimposed on stimulus) that occurred randomly across all stimulus categories, thereby avoiding category-related top-down effects of the task.

Consequently, the current study measured occipito-temporal N1 responses – as correlates of EBA activation^17,18^ – to images of hands vs chairs in distorted vs standard configuration, thereby using a factorial design (Fig. 1). We test the following predictions: (1) N1 responses to distorted hands will be stronger than to natural hands; and the difference between distorted and standard will be stronger for hands than for chairs; (2) the distortion-related response to hands (established in the distorted vs natural contrast) will be stronger in the right hemisphere (relative to left).

## Results

### Behavioural results

As EEG data acquisition was split into 5 runs, each subject reported 5 mental counts of stimuli with superimposed shadows: On average, mental counts were correct for 4 out of 5 runs (across 5 participants for whom data were available, see Methods). Erroneous counts included too low and too high counts. The 5 individual subjects’ total shadow counts were as follows: 53 (error + 1, relative to actual number of 52), 54 (±0), 62 (±0), 64 (−2) and 62 (−2), average error −0.6. Overall, these data suggest attentive performance on a task that was not too easy and unrelated to the stimulus categories of relevance to the research questions.

### ERP results

**Figure 2:**
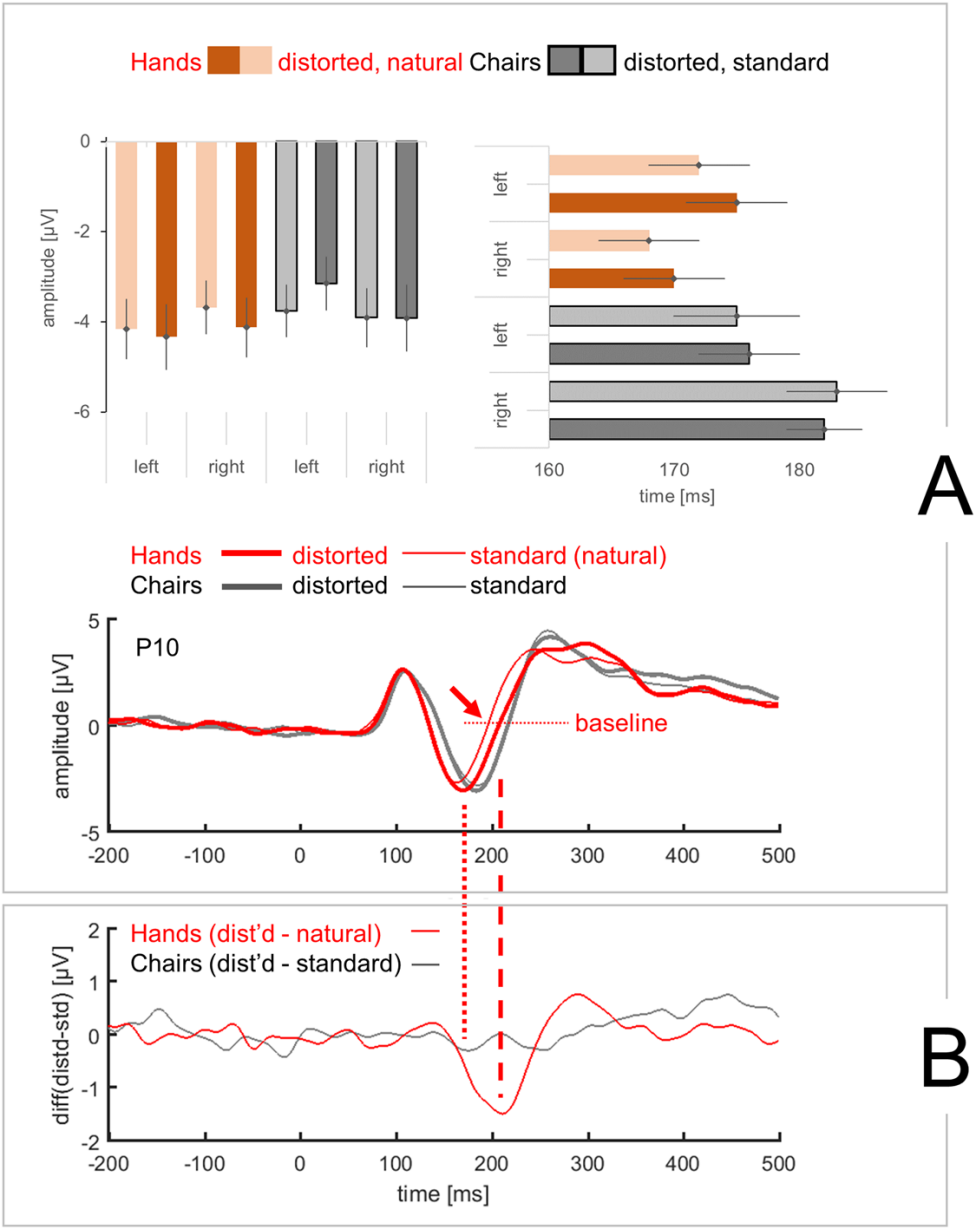
ERP amplitudes, latencies, and shape of N1 peak. (**A**) ERP time courses, N1 amplitudes and N1 latencies, separated by stimulus type (hands vs chairs), configuration (distorted vs natural) and hemisphere (left vs right). Upper left bar graph shows N1 amplitudes for each of the stimulus conditions (mean ± SEM). Along the y-axis, amplitude in µV. Upper right bar graph shows N1 latencies, separated as for amplitudes (mean ± SEM). Along the x-axis, latency in ms (relative to stimulus onset at 0 ms). In the xy-plot, time courses of ERPs are shown for hands (red) and chairs (black), each for distorted (heavy lines) and for standard configurations (thin lines). Grand averages across N = 14 participants, filtered 0.4–35 Hz. Stimulus onset at 0 ms. Along the y-axis, amplitude in µV. Negative peaks in 150–200 ms time window seen in all conditions. Arrows: For distorted hands, broader peaks (relative to natural hands). To allow sufficiently large display of peak shape, only one out of six electrode sites is shown. (**B**) Difference waveforms, distorted – natural hands and distorted – standard chairs, calculated from corresponding curves in panel A. Along the y-axis, amplitude in µV but scale different from panel B. Notice that the difference waveform peaks later than the per-condition waveforms in panel A. The peak of the difference waveform reflects a delayed return to baseline in the per-condition-waveform (dashed vertical lines). Note that the electrode site for the ERP time course example (P10) has been chosen to illustrate condition-related differences in the main analysis of N1 (Results, sections “N1 amplitudes”  and “Shape of the N1 peak”). At this example site, no stimulus-type-related P1 differences were seen, although such differences were observed in exploratory analysis of PO3 and PO4 data (Results, section “P1 amplitudes”).

Figure 2A shows ERPs from an example location (P10), illustrating stimulus-type- and configuration related ERP differences. To quantify these differences, we combined hypothesis-driven analysis of response strength at N1 latency with exploratory analysis in latency windows before and after N1. We studied amplitudes of P1 peaks (section “P1 amplitudes” below), amplitudes of N1 peaks (section “N1 amplitudes” below), the overall shape of N1 peaks in search of potentially prolonged responses (section “Shape of the N1 peak” below), and amplitudes in a broad window of 250 to 500 ms to capture response components later than N1 (Supplementary Results and Discussion).

#### P1 amplitudes (exploratory analysis)

P1 amplitudes were measured from electrodes PO3 and PO4 where P1 was clearly defined (Supplementary Table 1a). In ANOVA of these amplitudes, the main effect of stimulus type was significant (F(1,13) = 28.500, p < 0.001, η_p_^2^ = 0.687). Neither of the other main effects (configuration and hemisphere) was significant (see Supplementary Table 1b for complete ANOVA results). Out of the interactions, only stimulus type X hemisphere was significant (F(1,13) = 6.559, p = 0.024, η_p_^2^ = 0.335), with stronger left-hemispheric P1 responses to images of hands vs chairs (t(13) = 5.450, p < 0.001, d = 1.45; note that all hand images were of right hands). This result is in line with earlier reports of stimulus category effects at P1 latency, for example stronger responses to faces than to cars or to butterflies as reported and discussed in detail in earlier studies^11,14,24^. The P1 result is, however, not directly related to our research question and outside of the hypothesis-driven analysis. In the absence of configuration effects on P1. this result will not be discussed further.

#### N1 amplitudes (main analysis)

Measured in waveforms per condition (hands distorted, hands natural, chairs distorted, chairs standard) and averaged over 3 electrodes per hemisphere (P7, P9, PO7 and P8, P10, PO8), N1 amplitudes are given in Fig. 2A (upper left bar graph). Because responses to distorted stimuli were to be compared with responses to standard (natural) stimuli, the first analysis step was a t-test across standard stimuli only which compared hand vs chair responses (averaged across hemispheres). No differences were found (t(13) = −0.267, p = 0.794, Cohen’s d = −0.07), thereby justifying the choice of standard stimuli as a baseline for comparisons with distorted stimuli.

In the main ANOVA on N1 amplitudes, none of the main factors was significant (stimulus type, configuration, hemisphere, see Supplementary Table 2a). A significant stimulus type X configuration interaction (F(1,13) = 7.009, p = 0.020, η_p_^2^ = 0.350) reflected a distortion effect of opposite direction for hands vs chairs. A significant configuration X hemisphere interaction (F(1,13) = 13.879, p = 0.003, η_p_^2^ = 0.516) was based on distortion effects of opposite direction in left vs right hemisphere. No other interactions were significant (see Supplementary Table 2a for all other results of this ANOVA).

To facilitate the interpretation of the stimulus type X configuration interaction, two follow-up ANOVAs were performed. In the first of these ANOVAs, for hands only (Supplementary Table 2b), the only result with p below 0.1 was a main effect of configuration (F(1,13) = 3.624, p = 0.079, η_p_^2^ = 0.218, trend), reflecting higher-N1 amplitudes for distorted hands (Fig. 2A, upper left bar graph). In the second follow-up ANOVA, for chairs only (Supplementary Table 2c), the only significant result was a configuration X hemisphere interaction (F(1,13) = 5.859, p = 0.031, η_p_^2^ = 0.311), reflecting lower N1 amplitudes for distorted chairs in the left hemisphere (Fig. 2A, upper left bar graph) and not of immediate interest to the research question.

#### Shape of the N1 peak (main analysis)

To test for potentially prolonged responses to distorted stimuli (see example waveform in Fig. 2A), we compared the shape of N1 peaks across conditions, using difference waveforms. To separate prolonged responses from merely delayed responses without change of the peak shape, we measured the time of return to baseline after the N1 peak in per-condition waveforms. To interpret these times, we also measured N1 peak latencies as auxiliary parameters (which were not in themselves measures of interest for this study).

N1 latencies (Fig. 2A, upper right bar graph) were measured in P9 and P10, the electrodes with highest N1 amplitudes across stimulus types, configurations, and hemispheres. In ANOVA, the only significant results was a main effect of stimulus type (F(1,13) = 11.699, p = 0.005, η_p_^2^ = 0.474), combined with a significant stimulus type X hemisphere interaction (F(1,13) = 11.654, p = 0.005, η_p_^2^ = 0.473; all ANOVA results in Supplementary Table 3a). Accordingly, latencies were further analyzed in follow-up ANOVAs, split by stimulus type. For hands (Supplementary Table 3b), the only significant ANOVA result was a main effect of stimulus configuration (F(1,13) = 16.53, p = 0.001, η_p_^2^ = 0.560) due to responses to distorted configurations that were 2 ms slower than to natural configurations. For chairs (Supplementary Table 3c), the only ANOVA result with p below 0.1 was a main effect of hemisphere (F(1,13) = 3.197, p = 0.097, η_p_^2^ = 0.197, trend), as right- (vs left-) hemisphere responses were 7 ms delayed.

**Figure 3:**
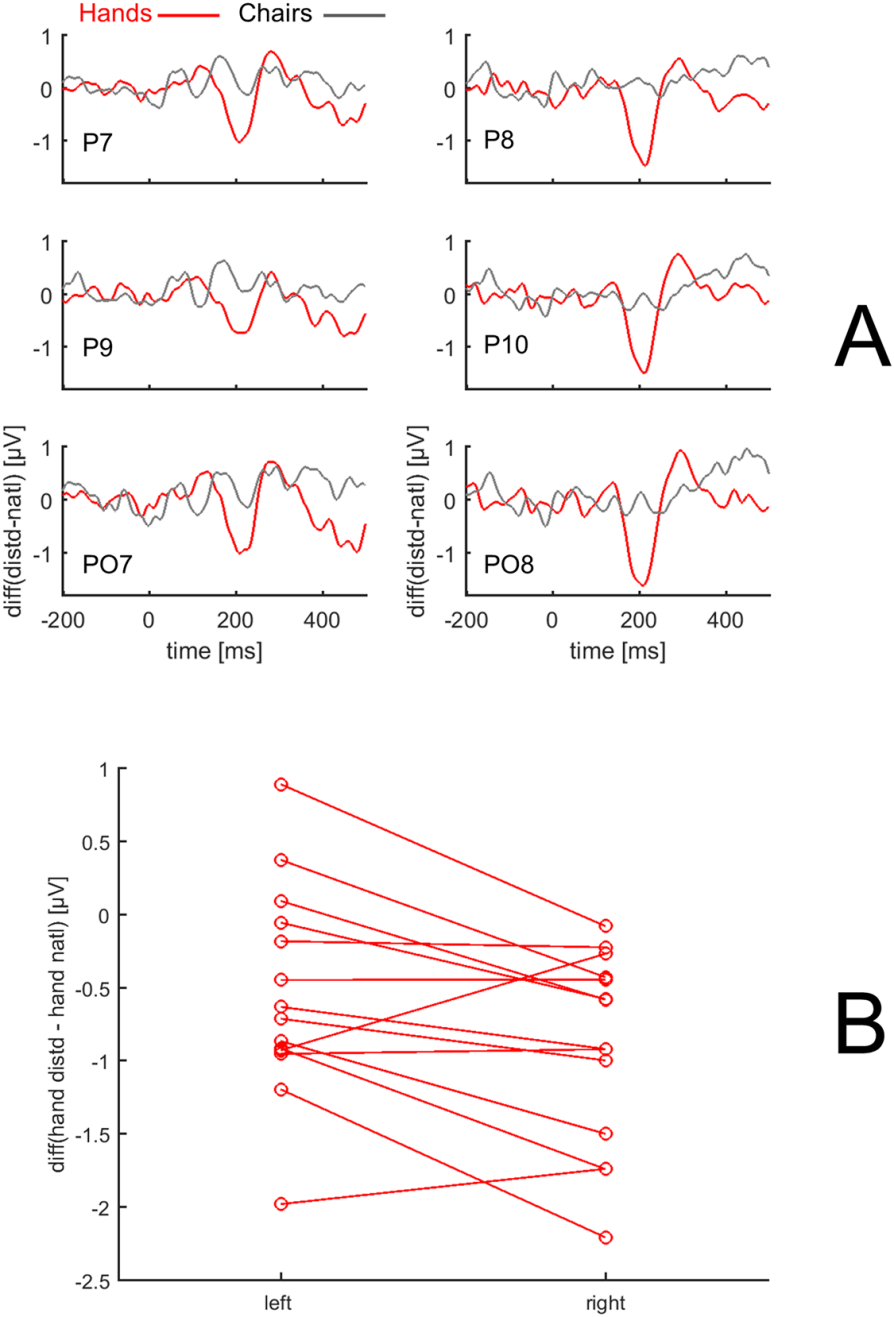
Analysis of difference waveforms. (**A**) Difference waveforms, distorted – natural for hands (red) and distorted – standard for chairs (black). Along the y-axis, amplitude difference in µV. Grand averages (N = 14) for electrodes P7, P9, PO7 (left hemisphere) and P8, P10, PO8 (right hemisphere). Across all electrodes, difference waveforms for hands – but not for chairs – show negative peaks in 150–250 ms latency range. (**B**) Peak amplitudes of difference waveforms for hands, averaged across 3 electrodes per hemisphere. For 9 out of 14 subjects, distorted – natural differences are larger (more negative) in right (relative to left) hemisphere, t(13) = 2.755, p = 0.016.

Difference waveforms (distorted – natural, example in Fig. 2B) served to identify potential condition-related differences in N1 response waveforms that were not captured in the analysis of peak amplitudes and latencies. Such difference waveforms were computed for hands, the condition of interest, and also for chairs as the control condition. Compare example time courses in Fig. 2A and B to see the additional information obtainable in difference waveforms. For each of the 6 electrodes, difference waveforms are shown in Fig. 3A, with clearly defined peaks for hands. In statistical analysis, peak amplitudes for hands (averaged across all 6 electrodes) were significantly different from zero (t(13) = −4.832, p < 0.01, d = −1.29). For chairs, no clear peaks were observable in difference waveforms (t(13) = 0.360, p = 0.725, d = 0.10). Consequently, further statistical analysis of difference waveform amplitudes was limited to the hands condition. In line with strikingly larger difference waveforms over the right (vs left) hemisphere (from visual inspection of Fig. 3A), a t test demonstrated robust hemispheric differences (t(13) = 2.755, p = 0.016, d = 0.74, amplitudes averaged across 3 electrodes per hemisphere). To illustrate the data underlying the grand averages in Fig. 3A and the statistical result, Fig. 3B shows subject-by-subject amplitudes of difference waveforms. Negative difference waveforms (indicating stronger negative-amplitude responses to distorted vs natural hands) were present for 11 out of 14 subjects over the left hemisphere and for 13 out of 14 subjects over the right hemisphere. Amplitudes were lower for right (vs left) hemisphere in 9 out of 14 subjects.

The difference waveforms, peaking ~40 ms later than N1 latency (see Fig. 2A,B), do not reflect N1 peak amplitude differences but rather changes in the shape of the N1 wave (for distorted vs natural hands). In the 3 right-hemisphere electrodes, the N1 peak for distorted hands was wider than for natural hands, with a later return to baseline (illustrated in grand averages of Fig. 2A for P10 example, see arrow). To establish whether this difference is statistically significant across the group of subjects, the time of return to baseline was measured from electrodes P9 and P10 where N1 peaks were best defined (see previous section and Methods). Times of return to baseline (as in Fig. 2A) were obtained in 11 out of the 14 subjects studied (in the remaining 3 subjects, no negative N1 amplitudes were found in baseline-to-peak measurement). One subject’s data were identified as outliers. ANOVA on the remaining 10 subjects’ data yielded a main effect of configuration (F(1,9) = 20.451, p = 0.001, η_p_^2^ = 0.694) and - as a trend - a configuration X hemisphere interaction (F(1,9) = 4.087, p = 0.074, η_p_^2^ = 0.312). For P9, the return to baseline was at 217 ± 10 ms (mean ± SEM) for distorted vs 212 ± 10 ms for natural hands (paired samples t-test: t(9) = 2.771, p = 0.022, d = 0.87). For P10, the return to baseline was at 202 ± 5 ms for distorted vs 193 ± 5 ms for natural hands (t(9) = 4.930, p = 0.001, d = 1.56). The delays for P9 and P10 indicate a prolonged response beyond what could be explained by the difference in peak latencies (distorted vs natural: 2 ms, see above). Consequently, at least for P9 and P10, a delayed return to baseline (in responses to distorted vs natural hands) underlies the peaks in difference waveforms (distorted – natural), observed bilaterally (Fig. 3A,B). The longer delay for P10 than for P9 (9 vs 5 ms, p = 0.074 for configuration X hemisphere in ANOVA above) is in line with larger difference waveform amplitudes for right vs left hemisphere (p = 0.016, see above). Thus, the difference waveforms indicate prolonged activation (broader negative peaks), in response to distorted (compared with natural) hands.

## Discussion

The current study found occipito-temporal ERP responses to salient images of distorted hands, manifest as stronger N1 (N170/190) responses, compared with N1 for natural hands. The modulation, seen as a trend only in per-condition response amplitudes, was revealed as statistically significant in difference waveforms, thereby comparing the entire shape of the N1 peaks. Four particular findings are noteworthy: (1) Distortion response varies with stimulus category: Stronger N1 responses to distorted stimuli were observed only for hands, as opposed to non-body-part images of comparable geometric structure and visual complexity. (2) Task demands: N1 modulation occurred in the absence of a distortion-related task. (3) Latency and peak shape: Modulation started remarkably early, from 170 ms post stimulus, affecting N1 as an ERP component that has been linked with activation of EBA. Distortion-related N1 modulation was visible as a broadening of the N1 peak, suggesting prolonged activation of EBA. (4) Laterality: N1 modulation for distorted vs normal hands was present over both hemispheres but strongly right-lateralized.

### Distortion response varies with stimulus category

The novel “distorted non-hand” control condition – modified from^7,8,23^ – helps to sharpen the interpretation of responses to distorted hands^1,3^. We used chairs rotated into an orientation where backrest and legs resemble back of the hand and fingers. Distorted chair legs corresponded to distorted fingers. Consequently, chair images were comparable to hands in geometrical configuration and complexity. However, for chairs the rotation resulted in an unfamiliar perspective, as opposed to the highly familiar first-person perspective for hands. Any effect of unfamiliar perspective would have been small, because subjects viewed a number of practice stimuli before the experiments, and all chairs were deliberately shown in a single perspective across 480 trials. In any case, it is important to note that – at least in our preselected electrode sites – there were no statistically significant amplitude differences between natural hands and standard chairs (t test for standard stimuli only, see Results). This finding supports the choice of chairs as suitable control stimuli for the purposes of this experiment.

Across hands and chairs, distorted stimuli were carefully matched in terms of how conspicuous the distortion was. Still, unlike distorted hands, distorted chairs were not associated with stronger N1 responses. Consequently, stronger responses to distortion, in the hand condition only, suggest neural circuits that are activated when distortion is seen in biologically relevant stimuli. Such circuits could underlie the high perceptual salience of distorted hands in social perception, as discussed in^1,3^.

Despite the good match between hand and chair stimuli, would it be possible that in the unfamiliar (rotated) chair stimuli subjects did not detect distortions as easily as in highly familiar hands? In that case one could expect similar distortion-related activity for chairs (as for hands) but weakened and/or delayed. However, N1 peaks did show sensitivity to distortion in chairs at the same latency as for hands but with different direction and topography (reduced responses in left hemisphere). Again, this result hints at circuits that are activated when distortion is detected in biologically relevant (hand) stimuli but not in chairs.

### Task demands

This study deliberately avoided any task-related attention to distortions. To perform the task, subjects had to be attentive across all stimulus categories as task-relevant stimuli were randomly distributed in the stimulus series, across all stimulus categories. Based on task demands and latency, the observed responses can be dissociated from responses to biologically impossible arm and hand postures occurring from 100 ms in deaf sign-language-proficient persons^25^. Such persons are highly trained in the observation of arm and hand gestures, unlike our participants.

### Latency and peak shape

Our results extend earlier studies such as an MEG study where distortion-related activation was found in bilateral occipital areas starting from 260 ms after stimulus^1^. Through measurement of a predefined ERP component, the current study reveals distortion-selective activation even before 260 ms, here seen as a modulation of N1 (N170/190). The latency is also consistent with modulated ERP responses at 190–230 ms latency when participants viewed hand or whole-body images with lower arm or thumb rotated into impossible positions - however, hand images were not analyzed separately in that study^26^. The body-sensitive N1 peak, targeted here through choice of electrodes and latency window, has been linked with EBA activation, based on ERP source analysis^17^ and comparison with fMRI data^18^. Our results, therefore, suggest a contribution of EBA to distortion-related processing. Consequently, as the modulation results in a broadened N1 peak, starting only after peak latency (Figs 2A,B and 3A), the distorted hands could have led to prolonged EBA activation. Could the distortion-modulated N1 be driven by feedback from the amygdala (activated in fMRI responses to distorted hands^3^)? Such feedback had been hypothesized for the distortion-related activity from 260 ms in Avikainen *et al*.^1^. Even at the early N1 latency, amygdalar feedback would be conceivable, given that at even earlier latencies (from 74 ms), amygdalar responses to socially relevant stimuli (fearful faces) have been observed in human intracranial data^27^. Also, amygdalar damage affects ERPs to fearful faces from 100 ms post-stimulus^28^. Amygdalar modulation of EBA responses to emotional whole-body movement has also been established using fMRI^29^.

### Laterality

N1 modulation, although present over both hemsipheres, was robustly right-lateralized. This finding is remarkable because all hands presented were shown as right hands for which a stronger left-hemisphere (contralateral vs ipsilateral) activation could be expected. The observed lateralization to the right is in line with our earlier fMRI study^3^. For right hands viewed in the first-person perspective (as in current study), the fMRI study found distortion-related sensorimotor activation with right-hemisphere dominance. In this respect, the current results – limited to a specific time window at N1 latency – complement the earlier fMRI findings. Furthermore, our right-lateralized N1 responses are in line with right-lateralized EBA activation in an earlier fMRI study^30^ with observation of videos showing impossible (vs possible) finger movement of the right hand. Right-lateralized EBA activation was also found for contorted whole-body postures, using fMRI^31^. The current N1 responses provide timing information that is lacking from these fMRI studies.

Given that distortion-related N1 modulation was found over both hemispheres (albeit with different strength), what could be underlying the right-lateralization of modulation? One possibility would be the demand for visuospatial processing as the subject constructs a three-dimensional representation of the observed hand from two-dimensional images. Visuospatial processing is known to be a right-hemisphere-dominant brain process (see for example^32^). However, right-hemispheric responses to control stimuli were not distortion-sensitive, although carefully matched for geometric complexity. Consequently, a better explanation of right-lateralized responses would be that images of hands – as biological stimuli – are emotionally laden. In earlier studies, subjects rated static images of distorted hands or videos of biologically impossible hand movement as unpleasant^3,30^, and negative emotional stimuli require right-hemisphere processing (reviews:^33,34^). The link between emotional contents and stronger N1 responses is tentative and requires further support, for example from an experiment that would include rarely seen, but not distorted hand postures in an additional condition. In the emotion-related interpretation, the right-lateralized N1 responses to distorted hands would be comparable to right-hemisphere modulation of N1 (190 ms latency) in a contrast of fearful vs neutral whole-body postures, presented as static images in an EEG study^21^. Correspondingly, right-hemisphere-only responses were found for fearful (vs neutral) body postures using MEG^35^. However, this response occurred at 80–110 ms (outside of our latency window for N1 analysis) and was localized to a parietal region distinct from EBA (outside of the area covered by electrodes analyzed in our study). Therefore the MEG results are not directly comparable with our findings although it is noteworthy that both studies used emotionally laden stimuli and found modulated responses in the right hemisphere. The aversive quality of distorted hands may induce a response that shares neural resources with processes of aesthetic judgment of body postures; such judgments are impaired after virtual lesions to right EBA^36^.

In conclusion, our results are in line with privileged visual processing of hands as highly salient body parts, with distortions engaging EBA resources that are especially activated for biological stimuli in social perception. Such activity was found from 170 ms after stimulus onset in a hypothesis-driven latency window and choice of electrodes, at an earlier stage than comparable MEG-measured responses from 260 ms in a previous study^1^. Although present over both hemispheres, distortion-modulated N1 peaks were right lateralized, suggesting an EBA processing step that co-occurs with – also right-lateralized – distortion-related activation in sensorimotor areas in fMRI^3^, interpreted as a correlate of embodied simulation^37^ within a network responding to observed bodily distortions^3^. Further studies are needed to establish if distortion-related EBA activation starts before sensorimotor activation (bottom-up processing), simultaneously with sensorimotor activation (potentially driven by a third site of activation), or after sensorimotor activation (as in a top-down process). Electrophysiological methods, as used in the current study, would be particularly useful to address these questions of activation timing in embodiment processes that contribute to social perception.

## Methods

### Subjects

A total of 15 subjects (11 female), of average age = 24.80 years (SD = 3.62) – all right-handed by self-report and with no history of neurological or psychiatric problems nor drug abuse, also by self-report – participated in this experiment. In preparatory analysis, data from one participant were removed as outliers (see below). Informed consent was obtained from all participants. The study was approved by the ethics committee of the School of Psychology (University of Nottingham) and performed in accordance with the declaration of Helsinki.

### Stimuli and procedure

Stimuli were images of 3D models of hands and chairs, computer-generated using Blender software (https://www.blender.org) according to an in-house protocol (developed by M.G.E.S., see Supplementary Information). Briefly, based on photos of actual hands, the procedure resulted in 48 images of hands (24 distorted, 24 standard, i.e. natural). Hand images were complemented with 48 images of chairs (24 distorted, 24 standard). Examples of stimulus images are shown in Fig. 1A.

Each of the stimuli was presented twice during a 192-image run of stimuli in pseudorandomized order, using Psychopy^38^. Each run took 5 min 20 s, and each subject completed 5 runs.

Stimuli were presented on a computer screen (ViewPixx3D, 1920 × 1080 pixels, 23,6”, 120 Hz refresh rate, 1ms pixel response time), viewed from approximately 60 cm. Stimuli were presented as 250 × 250-pixel images, resulting in a size of 4 degrees of visual angle, on a uniform grey background.

Images were presented for 200 ms, with an inter-stimulus interval of either 1000, 1200 or 1600 ms (randomly chosen), see Fig. 1B. In order to keep participants’ attention, a small number of images was superimposed with a white shadow (100 × 100 pixels, opacity ratio 0.6). The number of shadows that would appear was randomized between 8 and 16 per run. Subjects were informed of the minimal and maximal number at the beginning. They were  instructed to respond with a button press upon detection of a shadow and to keep a mental count of how many shadows they saw. Furthermore, before the start, they were shown example images for approximately two minutes of practice. At the end of each experimental run, they were asked how many shadows they counted. Although all subjects were asked, responses were only recorded for 6 subjects, of whom 5 were among the 14 subjects whose ERPs were entered into group level analysis. Out of these 5 subjects, 2 gave correct counts for each the 5 runs per subject, 1 subjects was correct for 4 runs, and 2 subjects were correct for 3 runs. Of the altogether 5 incorrect counts, 3 were too low and 2 were too high. A summary of these behavioural data is in the Results section.

### Data acquisition

Subjects were asked to sit in a Faraday-cage (2.5 × 2.3 × 2.3 m) quiet room at ambient temperature. EEG was recorded using a 64-channel Active-Two acquisition system (BioSemi, Amsterdam, Netherlands), sampled at 1024 Hz and digitized at 24-bits. 64 active electrodes were placed using a BioSemi cap, keeping impedances below 20 kΩ. An additional 6 Ag/AgCl electrodes were placed at M1, M2, F9, F10, T9, T10. Triggers were sent and received using a Cedrus Stimtracker. Data were collected using ActiView 7.0.

### Signal processing

Offline signal processing was performed in MATLAB (The MathWorks Inc., Natick, MA) using FieldTrip^39^ and EEGLAB^40^ routines. Due to active electrodes, BioSemi systems allow for reference-free recordings, and only at pre-processing stages, the time series of the electrodes were referenced to an all-electrode average. Data were band-pass filtered between 0.4 to 35 Hz followed by a visual search to identify obvious bad trials which were removed from the dataset. A plot trial by variance was created to identify which trials had the largest variance. Those that had standard deviations above 5 z-values were removed from the data. ERP data for each trial was collected, baseline corrected (−200 to 0 ms) and averaged for each participant, with an epoch length of −200 ms to 500 ms.

Segmented trials were then put through an independent component analysis (ICA) to remove blink, vertical, horizontal eye movements, and muscle activity related artefacts. Components containing artifacts were automatically flagged (Multiple Artifact Rejection Algorithm, MARA^41^). This algorithm is a linear classifier that rates different features of the topographies as noise or outliers and provides a classification of ‘reject’ or ‘accept’. This is a robust method (based on ICA) to identify components for muscle, blinks, eye movements, noisy channels. Still, if blinks (or other activity) are highly correlated with stimulus presentation it may classify the component as good. Therefore, the MARA step was complemented with visual inspection of the well-known topographies for artifacts to validate the classification.

### Event-related potential analyses

For each hemisphere, three electrodes overlying lateral occipito-temporal cortex were chosen for analysis: P7, P9, PO7 and P8, P10, PO8. This selection was based on an earlier study of N1 responses sensitive to images of whole bodies^17^. P7/P8 and PO7/PO8 were as in that study^17^. P9/P10 (neighbouring P7/P8) approximated two other electrodes in the previous study (PO9 and PO10) which were not available in the BioSemi cap.

In the first analysis step, per-condition waveforms were evaluated (distorted hands, natural hands, distorted chairs, standard chairs). N1 amplitudes were extracted based on the most negative waveform within a time period of 150 to 200 ms for each of the 6 electrodes, using a baseline of −200 to 0 ms. Amplitude measurements identified one participant’s data as outliers (outside 2 SD in multiple electrodes), therefore these data were removed from further analysis. The remaining 14 participants’ amplitude data underwent repeated measures ANOVA with within-subject factors stimulus type (hand, chair), configuration (distorted, standard), and hemisphere (left, right), using data averaged across 3 electrodes per hemisphere. The second analysis step started with ANOVA of N1 peak latencies with the same 3 within-subject factors as above, using data from P9/P10 as electrodes of maximal N1 amplitudes. Next, difference waveforms (distorted-standard) were analysed. Peak amplitudes of difference waveforms were measured in a time window of 150 to 250 ms (defined on the basis of visual inspection and therefore different from time window for latencies in per-condition waveforms, see Fig. 2A,B) against a baseline of −200 to 0 ms.

As measures of effect size, we calculated Cohen’s d for t-tests and η_p_^2^ for ANOVAS (see^42^ for review).

### Data availability

Data analyzed during the current study are available from the corresponding author on reasonable request.

## Electronic supplementary material

Supplementary Information 
