# Supplementary material for: Lateralized occipito-temporal N1 responses to images of salient distorted finger postures
Source: Sci Rep. 2017 Oct 26;7:14129. doi: 10.1038/s41598-017-14474-x (PMC5658422; doi:10.1038/s41598-017-14474-x)
Supplement: Supplementary file 1 — Supplementary Information [file 41598_2017_14474_MOESM1_ESM.pdf]

Lateralized occipito-temporal N1 responses to images of salient distorted finger postures

Miguel G. Espirito Santo, Hsin-Yuan Chen, Martin Schürmann

Supplementary Methods (page 1), Suppl' Tables 1-3 (p 2-4), Suppl' Results and Discussion (p 5-6)

Supplementary Methods

Hand stimuli were created using realistic 3D models of actual people's hands who participated in previous experiments conducted by the authors. In order to create these models, right hand dorsal and palmar sides were photographed (approximately 30-40 pictures each) from multiple angles, and then the pictures were uploaded separately onto Autodesk® 123D Catch. This free software allows photos to be uploaded, then through intensity mapping algorithms, creates a 3D mesh of the uploaded photos for each side of the hand. The models created through this software were then processed using Blender 3D®, a free 3D modelling software (https://www.blender.org). for 3D mesh post-processing and texture fixes.

Blender provides an API which allows easy access to edit any interfaced data and allowed applying distortions to the hands and chair stimuli consistently. For each of the hand models, the dorsal side with the wrist structure was parallel to the camera. Then the structure controlling the wrist was rotated by 1.5°, 1°, 8.3° (x,y,z) in order to mimic a similar perspective to that of viewing your own hand. A 40° rotation was applied anticlockwise along the x- and z-axis for each finger (except the thumb) to the proximal interphalangeal joint (between the proximal and intermediate phalanges) in order to mimic an abnormality as they might occur after an accident (see Figure 1A). Saturation was set to 0, contrast to 2.5, and brightness to 0.8, Then hands were rendered with Blender camera presets (12:11 aspect ratio) with a focal length 35 mm, and 800 x 800 pixels resolution. Using this procedure, 6 individuals' hands were modelled. Due to inter-individual differences in hand size and shape, manual adjustments were made in order to keep the ratio of hand to camera as similar between models as possible. Per hand, 8 images (2 per finger) of distorted finger postures were created. Out of these, 4 images (1 per finger) were randomly selected as stimuli for the experiment. For natural finger postures, 4 images of each hand were created. The whole procedure resulted in 48 images of hands.

Chair stimuli were created using a similar method through modification of 6 freely available templates (http://www.blendswap.com/blends/view/40140, user sizzler, license: CC-BY). Using Blender 3D, distortions were applied to the legs of the chair, in order to create a geometrically matched control for distorted hands As the legs of the chairs do not have any specific landmarks, the distortions were applied at 1/3 third of the leg proximally to the seating base (see Figure 1A) in order to mimic the placement of the distortions on the fingers. The chairs were placed in similar position as the hands, with legs - as control proxies for the fingers – pointing upwards in diagonal fashion, and the chair was rotated 45° in the z-axis to maximize viewing of all the legs. Using this procedure, 6 different chairs were modelled. The same script (including camera parameters) was used to create the distortions for each of the chairs, creating 8 distorted images (2 distortions for each of 4 legs). Out of these, 4 images (1 per leg) were randomly selected as stimuli for the experiment. For standard chairs, 4 images of each chair were created. The whole procedure resulted in 48 images of hands.

In order to hide the end of the hand model, as this may distract from the finger distortion, a Gaussian filter was applied to centre of the image such that the edges of the images, and consequently the end of model, were blurred while the centre of the image remained visible (see Figure 1A).

Supplementary Table 1a: P1 amplitudes, separate by stimulus type, configuration, and hemisphere (electrodes PO3 and PO4). The reasons to choose PO3 and PO4 for exploratory analysis of P1 responses were (a) clearly defined P1 responses in grand averages and (b) lowest variability across subjects (measured as SD, compared with P7/P8, P9/10, and PO7/PO8, the 6 electrodes of interest for N1 in main analysis). PO3/PO4 were also among the electrodes studied in Thierry et al. (2007).

**Stimulus type**	**Configuration**	**Hemisphere**	**Mean**	**SEM**
Hands	Distorted	Left (PO3)	4.042	0.415
		Right (PO4)	3.366	0.433
	Standard/Natural	Left (PO3)	3.706	0.304
		Right (PO4)	3.308	0.416
Chairs	Distorted	Left (PO3)	2.166	0.328
		Right (PO4)	2.530	0.467
	Standard/Natural	Left (PO3)	2.056	0.373
		Right (PO4)	2.845	0.467

Supplementary Table 1b: ANOVA, all conditions, on P1 amplitudes in per-condition waveforms (Supplementary Table 1a, above), all factors within-subject. Exploratory analysis.

	df	F	p	η_p^2
**stimulus type (hand, chair)**	**1,13**	**28.500**	**<0.001**	**0.687**
configuration (distorted, natural/standard)	1,13	0.135	0.719	0.010
hemisphere (left, right)	1,13	0.002	0.967	<0.001
stimulus type X configuration	1,13	1.208	0.292	0.085
**stimulus type X hemisphere (footnote 1)**	**1,13**	**6.559**	**0.024**	**0.335**
configuration X hemisphere (footnote 2)	1,13	3.435	0.087	0.209
stimulus type X configuration X hemisphere	1,13	0.162	0.694	0.012

Footnote 1: To facilitate interpretation of the stimulus type X hemisphere interaction, paired t tests were run on P1 amplitudes (all averaged across distorted and standard/natural conditions), with the following results: Hands PO3 (left hemisphere) vs PO4 (right hemisphere), $t(13) = 1.205$, $p = 0.250$, Cohen's $d = 0.32$; Chairs PO3 vs PO4, $t(13) = -1.203$, $d = -0.27$; PO3 hands vs chairs, $t(13)=5.450$, $p < 0.001$, $d = 1.45$; PO4 hands vs chairs, $t(13) = 2.142$, $p = 0.052$, $d = 0.57$. The latter two results, $p < 0.001$ and $p = 0.052$, reflect stronger P1 responses to hands than to chairs, with a particularly strong difference in PO3 (left hemisphere - note that all hand images shown were of right hands).

Footnote 2: To facilitate interpretation of the configuration X hemisphere interaction (observed as a trend), paired t tests were run on P1 amplitudes (averaged across stimulus types, hands and chairs), with the following results: Distorted PO3 vs Distorted PO4, $t(13) = 0.313$, $p = 0.759$, $d = 0.203$; Standard PO3 vs Standard PO4, $t(13) = -0.449$, $p = 0.661$, $d = -0.120$; Distorted PO3 vs Standard PO3, $t(13) = 1.151$, $p = 0.270$, $d = 0.308$; Distorted PO4 vs Standard PO4, $t(13) = -1.115$, $p = 0.285$, $d = -0.298$.

Supplementary Table 2a: ANOVA, all conditions, N1 amplitudes in per-condition waveforms (complementary with **Figure 2A, left panel**), all factors within-subjects.

	df	F	p	η_p^2
stimulus type (hand, chair)	1,13	1.582	0.231	0.109
configuration (distorted, natural/standard)	1,13	0.001	0.970	0.000
hemisphere (left, right)	1,13	0.020	0.889	0.002
**stimulus type X configuration**	**1,13**	**7.009**	**0.020**	**0.350**
stimulus type X hemisphere	1,13	1.760	0.207	0.119
**configuration X hemisphere**	**1,13**	**13.879**	**0.003**	**0.516**
stimulus type X configuration X hemisphere	1,13	0.583	0.459	0.043

Supplementary Table 2b: Follow-up ANOVA, hands only, all factors within-subjects.

	df	F	p	η_p^2
**configuration (distorted, natural/standard)**	**1,13**	**3.624**	**0.079**	**0.218**
hemisphere (left, right)	1,13	0.493	0.495	0.037
configuration X hemisphere	1,13	0.953	0.347	0.068

Supplementary Table 2c: Follow-up ANOVA, chairs only, all factors within-subjects.

	df	F	p	η_p^2
configuration (distorted, natural/standard)	1,13	2.586	0.132	0.166
hemisphere (left, right)	1,13	0.840	0.376	0.061
**configuration X hemisphere**	**1,13**	**5.859**	**0.031**	**0.311**

Supplementary Table 3a: ANOVA, all conditions, N1 latencies in per-condition waveforms (complementary with **Figure 2A, right panel**), all factors within-subjects.

	df	F	p	η_p^2
**stimulus type (hand, chair)**	**1,13**	**11.699**	**0.005**	**0.474**
configuration (distorted, natural/standard)	1,13	0.526	0.481	0.039
hemisphere (left, right)	1,13	0.094	0.764	0.007
stimulus type X configuration	1,13	1.065	0.321	0.076
**stimulus type X hemisphere**	**1,13**	**11.654**	**0.005**	**0.473**
configuration X hemiphere	1,13	0.711	0.414	0.052
stimulus type X configuration X hemisphere	1,13	0.170	0.687	0.013

Supplementary Table 3b: Follow-up ANOVA, N1 latencies, hands only, all factors within-subjects.

	df	F	p	η_p^2
**configuration (distorted, natural/standard)**	**1,13**	**16.532**	**0.001**	**0.560**
hemisphere (left, right)	1,13	2.638	0.128	0.169
configuration X hemisphere	1,13	0.656	0.432	0.048

Supplementary Table 3c: Follow-up ANOVA, N1 latencies, chairs only, all factors within-subjects.

	df	F	p	η_p^2
configuration (distorted, natural/standard)	1,13	0.017	0.897	0.001
**hemisphere (left, right)**	**1,13**	**3.197**	**0.097**	**0.197**
configuration X hemisphere	1,13	0.441	0.518	0.033

Supplementary Results and Discussion

Complementary to the hypothesis-driven analysis of N1 responses (with electrodes of interest and time window chosen on the basis of research literature), exploratory analysis was performed on responses to hands later than N1. The purpose of this analysis was to compare EEG responses in the current study with the bilateral MEG responses to distorted hands in an earlier MEG study (Avikainen et al. 2003). In the MEG study, differences between distorted and natural hands started at 260 ms after stimulus onset and were most consistent across subjects in a 400 to 600 ms time window. Consequently, the time window for EEG analysis was chosen to start at 250 ms and to end at 500 ms (end of segmented EEG trials).

In ANOVA of EEG amplitudes (averaged between 250 and 500 ms), neither of the main effects (configuration: distorted, natural; hemisphere: left, right) nor the configuration X hemisphere interaction was significant (Supplementary Table 4a).

The MEG parameter was source strength (always positive) whereas the EEG parameter was amplitude relative to baseline (positive or negative). Therefore, in an additional analysis step, EEG responses were assessed in terms of root-mean-square (rms) amplitudes (always positive). Again, in ANOVA of rms EEG amplitudes (250 to 500 ms), neither of the main effects (configuration; hemisphere) nor the configuration X hemisphere interaction was significant (Supplementary Table 4b).

Although the MEG study and the current EEG study have divergent results regarding post-N1 responses, this divergence cannot be interpreted as a difference of MEG vs EEG measurements, because there are more obvious explanations. The current EEG study was not designed to match the experimental conditions of the MEG study (stimulus duration 2 s, 15° x 17° of visual angle). Instead, we used parameters as in earlier EEG studies of N1 responses to whole bodies and body parts (here 200 ms duration and 4° x 4° of visual angle, compare with Thierry et al. 2006 - same duration, same size - and with Taylor et al. 2010 - duration 300 ms, same size). This approach allowed hypothesis-driven analysis, in line with our aim to enhance sensitivity for distortion-related responses at N1 latency (earlier than what was found in MEG), rather than replicating the MEG study. Note that in the MEG setup the time window of distorted vs natural differences is within stimulus duration whereas in the EEG setup, the 250 to 500 ms window is after stimulus offset.

Critically, the MEG study and our current study were also different in terms of the task for the subject: in the two conditions of the MEG study, subjects either detected stimulus repeats in a 1-back task or imitated the previously seen hand posture when an imperative (non-hand) stimulus was shown. In the current EEG study, subjects had to mentally count occurrences of a shadow superimposed on some of the stimuli. The MEG study therefore required processing of hand postures for demands of the 1-back and imitation tasks whereas in our study postures were entirely irrelevant to the task. It is all the more remarkable that our hand postures, although irrelevant to the task, still elicited distorted vs natural response differences at N1 latency (see Discussion, section "Task demands").

Supplementary Table 4a: ANOVA, hands only, mean amplitudes 250 to 500 ms, all factors within-subjects.

	df	F	p	η_p^2
configuration (distorted, natural/standard)	1,13	0.034	0.856	0.003
hemisphere (left, right)	1,13	1.642	0.222	0.112
configuration X hemisphere	1,13	1.019	0.331	0.073

Supplementary Table 4b: ANOVA, hands only, mean rms 250 to 500 ms, all factors within-subjects.

	df	F	p	η_p^2
configuration (distorted, natural/standard)	1,13	1.406	0.257	0.098
hemisphere (left, right)	1,13	0.966	0.344	0.069
configuration X hemisphere	1,13	0.014	0.909	0.001

References for Supplementary Material

Avikainen, S., Liuhanen, S., Schürmann, M. & Hari, R. Enhanced extrastriate activation during observation of distorted finger postures. J. Cogn. Neurosci, 15, 658-663 (2003).

Thierry, G. et al. An event-related potential component sensitive to images of the human body. NeuroImage 32, 871-879 (2006).

Thierry, G., Martin, C.D., Downing, P. & Pegna, A.J. Controlling for interstimulus perceptual variance abolishes N170 face selectivity. Nat. Neurosci. 10, 505-511 (2007).

Taylor, J.C., Roberts, M.V., Downing, P.E. & Thierry, G. Functional characterisation of the extrastriate body area based on the N1 ERP component. Brain Cogn. 73, 153-159 (2010).
